# Costs and Benefits Associated with the MRSA Search and Destroy Policy in a Hospital in the Region Kennemerland, The Netherlands

**DOI:** 10.1371/journal.pone.0148175

**Published:** 2016-02-05

**Authors:** Dennis Souverein, Patricia Houtman, Sjoerd M. Euser, Bjorn L. Herpers, Jan Kluytmans, Jeroen W. Den Boer

**Affiliations:** 1 Department of Epidemiology and Infection Prevention, Regional Public Health Laboratory Kennemerland, Haarlem, the Netherlands; 2 Department of Infection Control, Kennemer Gasthuis, Haarlem, The Netherlands; 3 Laboratory for Microbiology and Infection Control, Amphia Hospital, Breda, and University Medical Center, Utrecht, The Netherlands; University Medical Center Groningen, NETHERLANDS

## Abstract

**Objective:**

The objective of this study was to analyze the costs and benefits of the MRSA Search and Destroy (S&D) policy between 2008 and 2013 in the Kennemer Gasthuis, a 400 bed teaching hospital in the region Kennemerland, the Netherlands.

**Methods:**

A patient registration database was used to retrospectively calculate costs, including screening, isolation, follow-up, contact tracing, cleaning, treatment, deployment of extra healthcare workers, salary for an infection control practitioner (ICP) and service of isolation rooms. The estimated benefits (costs and lives when no MRSA S&D was applied) were based on a varying MRSA prevalence rate (up to 50%).

**Results:**

When no MRSA S&D policy was applied, the additional costs and deaths due to MRSA bacteraemia were estimated to be € 1,388,907 and 33 respectively (at a MRSA prevalence rate of 50%). Currently, the total costs were estimated to be € 290,672 (€ 48,445 annually) and a MRSA prevalence rate of 17.3% was considered as break-even point. Between 2008 and 2013, a total of 576 high risk patients were screened for MRSA carriage, of whom 19 (3.3%) were found to be MRSA positive. Forty-nine patients (72.1%) were found unexpectedly.

**Conclusions:**

Application of the MRSA S&D policy saves lives and money, although the high rate of unexpected MRSA cases is alarming.

## Introduction

Worldwide, infections with Methicillin Resistant *Staphylococcus Aureus* (MRSA) occur frequently in healthcare institutions [1]. In 2011, most European countries reported MRSA prevalence rates between 10% and 50%, while only six countries (Sweden, Norway, Iceland, Denmark, Estonia and the Netherlands) reported prevalence rates of less than 5% [2]. The most likely explanation for the low rate in the Netherlands is the MRSA ‘Search and Destroy’ (S&D) policy that was implemented in Dutch hospitals in 1988. This policy aims to prevent MRSA transmission and has resulted in stable and low MRSA prevalence rates since its introduction. Nowadays, the overall Dutch MRSA prevalence rate is estimated to be 1.7% (percentage of MRSA positive isolates with respect to all *Staphylococcus aureus* isolates) [3]. The Dutch MRSA S&D policy is characterized by active screening and pre-emptive isolation of high risk groups, as well as treatment of infections and colonization and isolation of MRSA positive patients in a single room until decolonization is successfully established [4].

In 2006, Bootsma et al. reported a significant association between application of the S&D policy and lower prevalence rates in hospitals and the community, which demonstrates its success in MRSA control [5]. In addition, several cost-benefit studies that were carried out in Dutch hospitals showed that the MRSA S&D policy is probably beneficial [6–8]. Despite these promising results, a major drawback of these studies is the lack of detailed information on costs and benefits of the separate components of the S&D policy, limiting the evaluation of these components. Several studies have suggested that minor changes (such as incorporation of PCR screening and excluding pre-emptive isolation in high risk groups from the MRSA S&D policy) may improve the costs of the S&D policy [9, 10]. Detailed information on costs and benefits of separate S&D policy components will help to evaluate the effect of adaptations of the policy.

Furthermore, the results of previously performed cost-benefit studies of S&D policies in the Netherlands may not be directly applicable to all regions. The regional MRSA prevalence can have a great influence on the estimation of costs of these policies [11]. For example, in some regions of the Netherlands the MRSA incidence increased three-fold since 2006, when persons who work with pigs and veal calves were added to the high risk group (33% of this group is colonized with livestock-associated MRSA (LA-MRSA)) [4, 7, 11]. In this perspective, regional cost-benefit studies are essential since regional differences in MRSA (and LA-MRSA) prevalence can influence costs and benefits involved with the S&D policy.

In the Kennemer Gasthuis, a 400 bed teaching hospital in the region Kennemerland, all actions involved with the MRSA S&D policy are registered by the Infection Control Department (ICD), allowing the possibility to evaluate the costs and benefits of this policy. The aim of the present study is to describe the epidemiology, costs and benefits of the MRSA S&D policy in the Kennemer Gasthuis, over a six-year period (between 2008 and 2013). Additionally, the total costs for inpatients were calculated for five different scenarios: (1) following the current guidelines whereby culture and PCR screening are used combined (as actually occurred), (2) following the current guidelines when only culture or (3) PCR screening was used and (4) a proposed adjustment scenario (abandoning pre-emptive isolation) using only culture or (5) PCR screening.

## Methods

### Ethics Statement

According to the Dutch regulation for research with human subjects, neither medical or ethical approval was required to conduct the study since the data were retrospectively recorded. Additionally we received approval to conduct the study from the institutional review board of the Kennemer Gasthuis which waived the need for participant consent. The data were anonymized and analyzed under code.

### The MRSA ‘Search and Destroy’ policy and population

The Dutch MRSA S&D policy protocol, which is implemented in the Kennemer Gasthuis, aims to actively search for MRSA positive patients through screening and isolation of (high) risk groups [4]. Risk groups can be divided into three groups: (1) high, (2) low and (3) no increased risk. High-risk groups are preventively isolated until microbiological test results are known, and isolation measures are continued when patients test positive and lifted when negative. These high-risk groups are: (1) microbiological confirmed MRSA positive patients, (2) patients who had unprotected contact with a MRSA positive person, (3) patients who were recently admitted to a hospital abroad (less than 2 months ago), (4) patients treated in a hospital with an ongoing MRSA outbreak, (5) foreign holiday dialysis patients and since 2006, (6) persons who work with pigs and veal calves. Low-risk groups are not nursed in isolation, but only tested for MRSA colonization (and isolated when testing positive) and defined as: (1) patients who were recently admitted to a hospital abroad (more than 2 months ago), (2) patients who had unprotected contact with a MRSA positive Health Care Worker (HCW) and (3) former positive patients (but not yet tested negative after one year). Patients without any of the above mentioned risk factors were assumed to be ‘not at risk’ for MRSA colonization. Patients who test positive receive decolonizing treatment and are considered decolonized when three consecutive negative tests are available with an interval of at least seven days. Apart from the risk approach described above, some patients have a clinical sample cultured with MRSA as an unexpected result during their hospital stay. In case of an unexpected MRSA positive patient, all contacts (patients and HCWs) are screened for MRSA colonization. Contacts (patients) are isolated until test results are known and measures are lifted when tests are negative. HCWs who test positive for MRSA are suspended from work and return to work after proven negative. Furthermore, when contacts (patients and HCWs) test positive, decolonizing treatment will follow according to the S&D policy. The ICD is responsible for consultation (for physicians, nursing personal and patients) and registration of the policy activities. This department also coordinates and performs the contact tracing (and the sampling of patients).

### Data collection

Between 2008 and 2013, data on the following items were collected by the ICD in the Kennemer Gasthuis: patient demographics, risk category (as described above), in-, out- or dialysis patient, MRSA positive or negative and if necessary, how many contacts (patients and HCWs) were screened. Additionally, admission data obtained from the hospital information system and culture data obtained from the laboratory information system of the Regional Public Health Laboratory Kennemerland (RPHLK) were combined with the MRSA screening database.

### MRSA screening procedures

At admission, all patients were interviewed and categorized in risk groups. For high and low risk groups screening was performed with either the PCR (when the patient needed an invasive procedure) or culture method (for all other patients). For inpatients and contacts, a MRSA screening set consisted of three (throat, nose and perineum) swabs [12]. For out- and dialysis patients, a MRSA screening set consisted of two swabs (throat and nose) and one swab (nose) respectively. To limit the burden in the outpatients clinic and for privacy reasons we choose for this culture regime during the study period. When positive, only one positive swab per set was completely characterized. After treatment, a MRSA follow-up test consisted of three negative MRSA sets of three swabs each (throat, nose and perineum). The average time between date of sampling and test outcome for culturing and PCR was considered to be five and one day(s) respectively.

### Isolation, treatment of carriers and other additional procedures

Personal Protective Equipment (PPE) was used for nursing the patient in strict isolation as part of the MRSA S&D policy [4]. A complete set of PPE consisted of gloves, a gown and a N95 respiratory mask (covering nose and mouth). Based on observations of the ICD, on average three sets of PPE were used for out- and dialysis patients, per patient, per day. For inpatients, on average fifteen sets each and additionally, twenty extra pairs of gloves and masks were used per patient on a daily basis. For intensive care (IC) patients, 0.5 FTE extra HCWs were employed per isolation day.

MRSA carriers were treated with rifampicin (twice daily 600 mg, seven days) and an antibiotic treatment following the antibiotic resistance pattern (such as trimethoprim-sulphametoxazole, twice daily 960 mg, seven days or doxycycline, daily 200 mg, seven days) until eradication was achieved [13]. As part of the eradication treatment, betadine shampoo (75 mg/ml) on day 2 of the treatment, mupirocin ointment (three times a day, five days) and chlorhexidine soap (daily 40 mg/ml, during the complete treatment) were used for disinfection of hair, nose and skin.

For positive inpatients, a daily additional disinfection and room cleaning procedure was performed as well as after discharge. For positive IC patients, one extra cleaning was incorporated.

### Variable costs

#### MRSA culture and PCR costs

Swabs were analyzed following the Standard Operating Procedure (SOP) for detection of MRSA at the RPHLK, which is based on the national guideline of the Dutch Association of Medical Microbiology (NVMM) [12]. Laboratory costs included: culture media, personnel and depreciation of laboratory equipment.

The following costs were related to culture and PCR:

- Culture: € 16.
- PCR (PCR costs + culture): € 91 (€ 75 + € 16).

Additional costs (in case of a positive culture or PCR):

- Species determination (MALDI-TOF): € 6.
- Vitek2 susceptibility testing: € 49.
- Confirmation (detection of the Martineau and *mecA* gene): € 50.

The confirmation step of MRSA suspected isolates consisted of (1) the detection of the Martineau gene, which is specific for *Staphylococcus aureus* isolates and (2) the *mecA* gene which codes for the penicillin binding protein 2 (PBP2A) that is resistant towards methicillin.

The culture and PCR set costs (positive and negative) for all patient groups are shown in Table 1. Additionally, costs for PPE, treatment, additional room cleaning, extra IC nursing and costs for hiring substitute HCWs for one week are also shown in Table 1.

**Table 1:** Variable costs.

Culture/PCR costs ^ω^:	Inpatients	Outpatients	Dialysis
Culture(s) pos (set)	€ 153	€ 137	€ 121
Culture(s) neg (set)	€ 48	€ 32	€ 16
PCR pos (set)	€ 378	€ 287	€ 196
PCR neg (set)	€ 273	€ 182	€ 91
Follow-up set	€ 144	€ 144	€ 144
**Use of PPE (set)** ^**¥**^:	€ 18	€ 3	€ 3
**Other variable costs (for all groups):**			
Treatment ^α^	€ 45		
Cleaning ^Ψ^	€ 50		
Week of for positive HCW	€ 830		
Extra HCW for IC nursing ^β^	€ 87		

ω Screening/follow-up costs represent set costs; For in-, out- and dialysis patients: set consists of three, two and one swab(s) respectively; When positive, only one swab was completely characterized. Follow-up swabs consists of three times a set of negative cultures.

¥ Use of PPE consists of a set; For inpatients: set consist of 35 masks and gloves and 15 gowns per day; For outpatients and dialysis patients: set consist of three masks, gloves and gowns.

α Treatment consists of antibiotics (seven days), betadine shampoo, mupirocine ointment and chloorhexidine soap for disinfection of hair, nose and skin.

Ψ Additional cleaning costs per isolation day.

β Additional nursing costs per isolation day (0.5 FTE) for IC patients.

### Fixed costs

Defined fixed costs included annual service of isolation rooms (service and replacement of the hepa filter) and annual salary cost for 0.15 FTE infection control practitioner (ICP). These 0.15 FTE contains only time that is specifically devoted to the MRSA S&D policy (like contact tracing for unexpected positive patients, registration of MRSA S&D data, consultation and management of protocols). In the Kennemer Gasthuis, no additional costs were made for building new isolation rooms during the study period and are therefore not included in the cost calculation. For nursing MRSA positive patients 25 isolation rooms were available of which three were built (before the study period) following the latest guidelines (with a negative are pressure gradient of 7.5 Pascal) [14]. The other 22 isolation rooms (specified following the old directive, with a negative air pressure gradient of 2–4 Pascal combined with a hepa filter) were evenly divided over all other nursing wards and also available for nursing MRSA positive patients. Additionally, a percentage of ten percent for unknown costs (such as: extra culture costs for patients with wounds) was taken into account.

### Total costs calculation between 2008–2013

The total costs from the hospital point of view as actually occurred for all patient groups (in-, out- and dialyze patients), using PCR and culture screening combined, were used as main outcome and compared to the benefits calculation. Additionally, the total costs for inpatients were calculated for five different scenarios: (1) following the current guidelines whereby culture and PCR screening were used combined (as actually occurred), (2) following the current guidelines when only culture or (3) PCR screening was used and (4) a proposed adjustment scenario (abandoning pre-emptive isolation) using only culture or (5) PCR screening. All calculated costs were based on the accepted costs in 2013.

### Cost calculation abandoning pre-emptive isolation in high-risk inpatients

Based on the proposed adjustment of Wassenberg et al., a cost calculation was made abandoning pre-emptive isolation of high-risk inpatients since colonization and chance of transmission was low in high-risk inpatients [9]. They showed that only 1.6% of the inpatients hospitalized abroad and 3.2% of all high-risk inpatients were colonized with MRSA. This means that 96.8% of these inpatients were unnecessarily nursed in isolation. Based on Wassenberg’s adjustment, we made a calculation of the expected costs using only PCR or culture screening after adjustment. An assumption was made that the previously expected positive patients were now unexpected (whereby contact tracing is indicated), since these inpatients were not isolated from the start of admission. We assumed that using the PCR screening method, 66% less contacts were screened since in our hospital PCR screened inpatients were on average 2.3 days shorter isolated than culture screened patients.

### Benefits

In order to estimate the benefits of the S&D policy, the number of prevented patients was compared to a hypothetical situation in which the S&D policy was never applied. The assumptions for such a situation have been published before by van Rijen et al. [7]. Van Rijen et al. assumed that 50% (prevalence) of the total nosocomial *S*. *aureus* bacteraemia (SAB) patients would have been caused by MRSA and that these patients are additional to the existing number of nosocomial SAB, as described for many countries that do not apply such a MRSA S&D policy [2, 15]. For our costs calculation, we used the median hospital costs for patients with a MRSA bacteraemia of Cosgrove et al., which were estimated on € 11.871 per patient [16]. In order to calculate the number of patient lives saved by implementation of the S&D policy, the mortality rate of SAB as presented by van Rijen et al. was used, which was 28.4% [7]. No difference in mortality rate between MRSA and susceptible *S*. *aureus* was taken into account.

The annual number of patients with a nosocomial *S*. *aureus* bacteraemia (SAB) who were diagnosed in the Kennemer Gasthuis were used to calculate the number of MRSA bacteraemia patients. Costs and deaths were calculated by applying the assumptions of van Rijen et al. and Cosgrove et al. to our population. In our study, patients with a nosocomial SAB were identified using the laboratory information system of the RPHLK. For each patient, per year the first *S*. *aureus* positive blood sample was included (2008 to 2013). A bacteraemia was defined as nosocomial when the blood sample was cultured more than two days after admission.

### Data analysis

All patients registered in the MRSA database were categorized based on year of consultation, location of consultation (in-, outpatient or dialysis center) and MRSA screening test outcome (if tested positive, with or without nosocomial spread). Nosocomial spread was inferred from contacts who tested positive for MRSA as part of contact tracing (molecular typing results were not taken into account). The average costs and benefits were calculated between 2008–2013 and specified per patient group, using the costs from the current situation as actually occurred (when PCR and culture was used combined). In addition, a break-even point was calculated by varying MRSA prevalence rates. Calculations were performed with Microsoft Excel 2013 and PASW Statistics/SPSS version 18.0.

## Results

Between 2008 and 2013, a total of 1712 patients and HCWs (285 annually) were subjected to the MRSA S&D policy, of whom 1087 (63.5%) patients and HCWs were part of contact screening (Table 2). Five hundred seventy-six patients were screened because they were categorized as high-risk group (following the Dutch national protocol) of whom 19 patients were found positive (3.3%) (Table 3). The majority of the screened patients (544 patients, 94.4%) was previously hospitalized abroad. From these patients, 11 patients (2.0%) were found MRSA positive. All together (without contact tracing results), 68 patients (screening and unexpected positive) were found to be MRSA positive of whom 49 (72.1%) patients were not categorized as high-risk and therefore unexpected. All these unexpected patients were tested based on clinical indications, generally because of wounds.

**Table 2:** Number of patients per category between 2008–2013.

	Inpatients	Outpatients	Dialyze patients	Subtotal	Contact screening (patients and HCWs)	Total
**MRSA screening negative**	185	193	179	557	1074	1631
**MRSA screening positive**	9	10	0	19	13	32
**Subtotal**	194 (33.7%)	203 (35.2%)	179 (31.1%)	576 (100%)	1087	1663
**Unexpected positive**	23	26	0	49	-	49
**Total**	217	229	179	625	1087	1712
**Annual average**	36	38	30	104	181	285

**Table 3:** MRSA prevalence in risk groups between 2008–2013.

MRSA risk category	At risk	Positive (%)	Inpatients	Outpatients	Dialyze
**All MRSA screened patients (at admission)**	576	19 (3.3%)	9/194(4.6%)	10/203 (4.9%)	0/179 (0%)
**Recently hospitalized abroad (at admission)**	544	11 (2.0%)	2/172 (1.2%)	9/195 (4.6%)	0/177 (0%)
**Other risk factors (at admission)** **^**	24	8 (25%)	7/22 (31.8%)	1/7 (12.5%)	0/2 (0%)
**Part of contact tracing (patients and HCWs)**	1087	13 (1.2%)	11/890 (1.2%)	2/197 (1.0%)	0/0 (0%)

^ Such as: known MRSA positive, in contact with a MRSA positive patient or family member, MRSA positive in the past and veterinarian.

### Total costs

**Fig 1:**
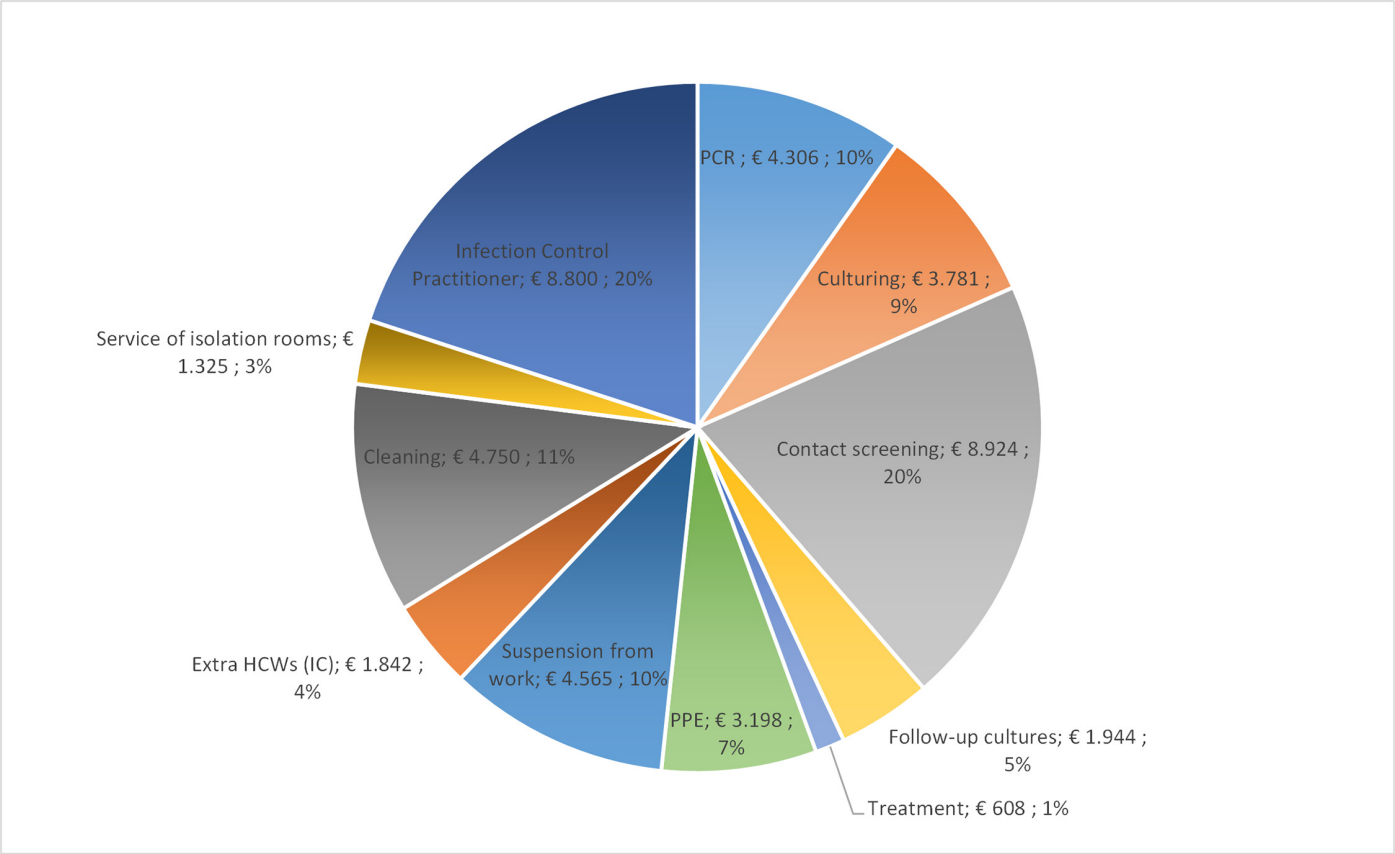
Annually average costs per category for the MRSA Search and Destroy policy in the Kennemer Gasthuis, Netherlands.

The total cost of the MRSA S&D policy in the Kennemer Gasthuis was € 290,672 between 2008 and 2013, which is on average € 48,445 per year. For in-, out- and dialyze patients the annually costs were € 28,229, € 5,066 and € 621 respectively (Table 4). Additionally, Fig 1 shows the annual average costs separated per item of the S&D policy and indicates that the main costs (€ 18,955 (44%)) can be attributed to screening, contact tracing and follow-up, including the costs for culture and PCR.

**Table 4:** Costs of the MRSA Search and Destroy policy between 2008 and 2013.

	Inpatients			Outpatients			Dialyze patients			Annual costs	Total costs
**Variable costs:**	**Number**	**Costs**	**Total**	**Number**	**Costs**	**Total**	**Number**	**Costs**	**Total**		
Un- and -expected patients (PCR pos)	5	€ 378	**€ 1.890**	1	€ 287	**€ 287**	0	€ 196	**€ 0**	**€ 363**	**€ 2.177**
Un- and -expected patients (PCR neg)	78	€ 273	**€ 21.294**	11	€ 182	**€ 2.002**	4	€ 91	**€ 364**	**€ 3.943**	**€ 23.660**
Un- and -expected patients (culture pos)	27	€ 153	**€ 4.131**	35	€ 137	**€ 4.795**	0	€ 121	**€ 0**	**€ 1.488**	**€ 8.926**
Un- and -expected patients (culture neg)	107	€ 48	**€ 5.136**	182	€ 32	**€ 5.824**	175	€ 16	**€ 2.800**	**€ 2.293**	**€ 13.760**
Follow-up cultures	32	€ 144	**€ 4.608**	36	€ 144	**€ 5.184**	0	€ 144	**€ 0**	**€ 1.632**	**€ 9.792**
Screening of contacts (pos)	11	€ 153	**€ 1.683**	2	€ 153	**€ 306**	0	€ 153	**€ 0**	**€ 332**	**€ 1.989**
Screening of contacts (neg)	879	€ 48	**€ 42.192**	195	€ 48	**€ 9.360**	0	€ 48	**€ 0**	**€ 8.592**	**€ 51.552**
Follow-up cultures contacts	11	€ 144	**€ 1.584**	2	€ 144	**€ 288**	0	€ 144	**€ 0**	**€ 312**	**€ 1.872**
Treatments (contacts, patients and HCWs)	43	€ 45	**€ 1.935**	38	€ 45	**€ 1.710**	0	€ 45	**€ 0**	**€ 608**	**€ 3.645**
PPE use (isolation days for inpatients)	999	€ 18	**€ 17.982**	203	€ 3,15	**€ 639**	179	€ 3,15	**€ 564**	**€ 3.198**	**€ 19.185**
Weeks of positive HCWs	33	€ 830	**€ 27.390**	0	€ 830	**€ 0**	0	€ 830	**€ 0**	**€ 4.565**	**€ 27.390**
Extra HCWs on IC (number of days)	127	€ 87	**€ 11.049**	X	X	X	X	X	X	**€ 1.842**	**€ 11.049**
Cleanings	570	€ 50	**€ 28.500**	X	X	X	X	X	X	**€ 4.750**	**€ 28.500**
**Total variable costs:**			**€ 169.374**			**€ 30.395**			**€ 3.728**		**€ 203.497**
**Annual average variable costs:**			**€ 28.229**			**€ 5.066**			**€ 621**		**€ 33.916**
										** **	
**Fixed costs:**										** **	** **
Infection control practitioner (0.15 FTE) ^ψ^										**€ 8.800**	**€ 52.800**
Service of isolation rooms ^≠^										**€ 1.325**	**€ 7.950**
**Total fixed costs**											**€ 60.750**
**Annual average fixed costs:**											**€ 10.125**
										** **	** **
**Subtotal variable + fixed costs**											**€ 264.247**
**Overheads 10%:**											**€ 26.425**
**Total costs:**											**€ 290.672**
**Total annual costs:**											**€ 48.445**

ψ One infection control practitioner spend on average five full weeks per year on the MRSA S&D policy.

≠ Service of isolation rooms comprise change and testing of the hepa filter.

Inpatients were nursed in isolation for a total of 999 days (167 days on average each year) which reflects on average € 170 additional costs per isolation day, per inpatient (total costs of inpatients divided by the total number of isolation days). In addition, Tables 5 and 6 shows the number of patients and costs per category of in- and outpatients.

**Table 5:** Costs per category of inpatients.

	Number of inpatients	Number of isolation days	Subtotal of variable costs	Mean variable costs per isolation day	Mean variable costs per patient
**Inpatient: screening negative**	185	456	€ 39.075	€ 86	€ 211
**Inpatient: screening positive**	9	75	€ 14.654	€ 195	€ 1.628
**Inpatient: unexpected positive (without nosocomial spread)**	17	346	€ 56.082	€ 162	€ 3.299
**Inpatient: unexpected positive (with nosocomial spread)**	6	118	€ 58.957	€ 500	€ 9.826
**Total inpatients**	217	995	€ 168.768	€ 170	€ 778

**Table 6:** Costs per category of outpatients.

	Number of outpatients	Subtotal of variable costs	Mean variable costs per patient
**Outpatient: screening negative**	193	€ 8.284	€ 43
**Outpatient: screening positive**	10	€ 3.442	€ 344
**Outpatient: unexpected positive (without nosocomial spread)**	26	€ 17.020	€ 655
**Outpatient: unexpected positive (with nosocomial spread)**	0	€ 0	€ 0
**Total outpatients**	229	€ 28.745	€ 126

The total costs for screening and isolating (positive) high-risk inpatients (without unexpected positive inpatients) in the current situation (using both culture and PCR) were estimated to be € 53,729 (€ 8,955 per year). When only PCR or culture would be used, these costs were estimated to be € 68,045 (€ 11,341 per year) or € 41,096 (€ 6,849 per year) respectively. Although the isolation costs using only PCR were lower compared to culture (€ 13,053), the laboratory costs were higher for PCR compared to culture (€ 43,650).

### Cost calculation abandoning pre-emptive isolation in high risk inpatients

If pre-emptive isolation would be abandoned in the Dutch MRSA S&D policy (as proposed by Wassenberg et al.[9]), a total of 456 isolation days could be averted, resulting in lower isolation costs for both PCR (€ 5,403) and culturing (€ 18,456). As a consequence of the proposed adjustment, nine non-isolated positive patients (for whom costly contact tracing is indicated) would have been identified. In our hospital, 26% (6/23) of the unexpected (non-isolated) MRSA cases (inpatients) showed nosocomial spread (to patients and HCWs) resulting in two and seven patients with and without nosocomial spread. This leads to higher costs (in case of PCR € 2,599 and culture € 6,031) for this patient group since contact tracing is indicated compared to a situation in which they would have been properly isolated. When comparing the total costs of the situation with and without pre-emptive isolation, the situation without pre-emptive isolation would save costs for both the PCR (€ 2,805) and culture (€ 12,425) scenario resulting in a total screening and isolation cost of € 65,240 using PCR against € 28,671 using culturing.

### Benefits

Between 2008 and 2013, 117 patients developed a nosocomial SAB (20 patients per year) resulting in an incidence density of 2.2/10,000 patient days (117/527,267). When a MRSA prevalence rate of 50% was taken into account, 50% of the total nosocomial SAB positive patients were caused by MRSA. Assuming that all these MRSA positive bacteraemia patients are additional to the current SAB burden, 117 additional MRSA bacteraemia patients would be found. In this situation, the total costs/savings without a MRSA Search and Destroy policy would be € 1,388,907 (117 times € 11.871) which is on average € 231,485 euro per year. Assuming comparable MRSA and MSSA mortality rates, this would result in 33 additional deaths (6 per year).

**Fig 2:**
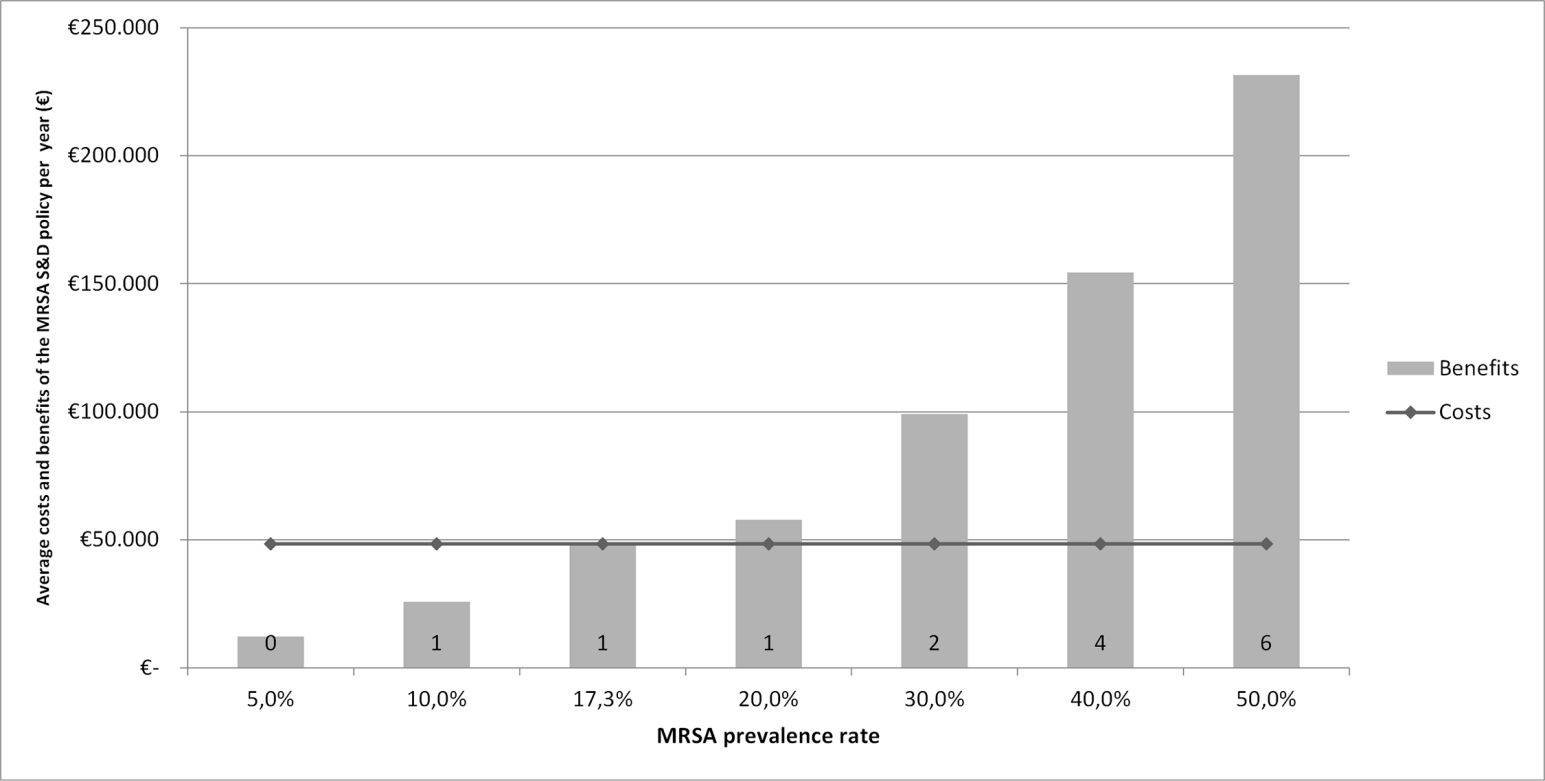
Annual costs and benefits of the MRSA S&D policy with a changing MRSA prevalence rate. The number above the MRSA prevalence rate describes the number of additional deaths per year at the associated prevalence rate.

Fig 2 shows the average costs and benefits (including the number of additional deaths) per year with a changing MRSA/MSSA prevalence rate (and 100% addition). In our study, a MRSA prevalence rate of 17.3% was considered as ‘break-even point’. In comparison, the Dutch MRSA prevalence rate in 2013 was 1.7% [3].

## Discussion

For this present study we analyzed the costs and benefits of the MRSA S&D policy in the Kennemer Gasthuis, a teaching 400 bed hospital in the region Kennemerland, the Netherlands. From our data it was calculated that the MRSA S&D policy did not only save six lives per year, but also costs (at a MRSA prevalence rate of 50%). We expect that this is an underestimation, since higher MRSA mortality rates were reported in relation to MSSA (34% against 27%) [15]. In our study, a MRSA prevalence rate of 17.3% is considered as break-even point. In fact, countries that do not have a S&D policy report MRSA prevalence rates up to 50% [2]. This means that abandoning the MRSA S&D policy (and increasing MRSA prevalence rates) is associated with higher costs than continuing the MRSA S&D policy.

In total, the costs for the MRSA S&D policy between 2008 and 2013 were estimated to be € 290,672 (€ 48,445 per year). Earlier, three other Dutch studies calculated the annual MRSA S&D costs in three different hospitals which resulted in the following costs: € 215,559 (van Rijen et al., 2009), € 1,383,200 (Nulens et al., 2008) and € 280,000 (Vriens et al., 2002) [6–8]. When the S&D costs were standardized per 100 patients per year the costs were estimated to be € 65,466 (Kennemer Gasthuis), € 90,953 (van Rijen et al.), € 562,276 (Nulens et al.) and €92,409 (Vriens et al.). A comparison between our study and these three previous studies shows several discrepancies, which may explain the differences in standardized estimated annual costs. First, in the Kennemer Gasthuis no revenue loss occurred since no new admissions were stopped and no surgical procedures were avoided because no major outbreaks had occurred. All outbreaks were manageable since no more than one secondary patient (who was still admitted) was identified among a single index patient. When contact tracing, following the MRSA S&D policy was performed among an unexpected MRSA positive patient, and more than one secondary positive patient was found, the Outbreak Management Team (OMT) of the Kennemer Gasthuis decided about closing wards and/or stopping new admissions. The OMT has considered this several times during the study period, but it never resulted in an admission stop. This policy is in line with the Dutch national MRSA S&D guidelines where it is stated that the OMT makes a decision about closing wards based on the hospitals patient population and consequences when ongoing transmission is detected. Second, the additional daily isolation costs were much lower in the Kennemer Gasthuis, since no additional HCWs were deployed (on normal nursing wards) for isolated inpatients. For example, Nulens et al. calculated € 406 of isolation costs per isolation day, mainly caused by deployment of extra HCWs. In the Kennemer Gasthuis, the isolation costs were € 18 and € 105 for normal and IC nursing wards. Third, the fixed costs in the Kennemer Gasthuis were much lower since no new isolation rooms (with a negative air pressure gradient of 7.5 Pascal) were built and 0.15 FTE ICP was calculated, instead of 1 FTE by van Rijen et al. Fourth, the prevalence of livestock associated MRSA is low in the region Kennemerland, in comparison to other regions resulting in a lower MRSA incidence rate [11]. All together, these discrepancies may explain the difference in costs between the present and the three earlier studies, and emphasize the relevance of performing cost-benefit studies of MRSA S&D policies in different hospitals, even in a small country like the Netherlands were a widely supported policy is implemented.

As shown in Table 5, the average additional isolation costs per inpatient were considerably higher for the unexpectedly positive patient group. In this perspective, it is essential to adequately identify and isolate high-risk patients to save costs for contact tracing. However, it is alarming to see that 72.1% of the MRSA positive patients had no risk factors, showing that infections and colonization were more frequently found in non high-risk patients. This emphasizes the importance of good basic hygiene (such as maintaining hand washing protocols) that prevents transmission of MRSA (or other MDROs) from unknown positive patients within institutions. When patients are proven MRSA and multi resistant *Acinetobacter* spp. negative, strict isolation measures were changed to contact isolation until patients are proven MDRO negative (which is advised for the other MDROs), instead of lifted (as happened before) [17]. These other MDRO tests are routinely done by culturing (which is more time consuming compared to MRSA PCR). The proposed cost savings (daily isolation costs) using MRSA PCR have decreased after this guideline change, since patients are still isolated in strict isolation (if *Acinetobacter* spp. result is not yet available) or contact isolation (awaiting for all other MDRO screening results). As shown in our analyses, no cost savings were demonstrated using MRSA PCR instead of culturing. Faster but above all cheaper diagnostics for all MDROs are needed to save (isolation) costs.

The main characteristic that determines the success of programs like the Dutch MRSA S&D policy is a widely supported policy that is feasible. In this context Wassenberg et al. suggested to lift pre-emptive isolation and only isolate patients when they test positive by using fast detection techniques such as PCR and/or chromogenic media [9]. This proposed adjustment might save costs and unnecessary isolation of patients and thereby strengthen the feasibility and success of the S&D policy in the future [9]. Our data suggest that when only PCR or culture screening would be used, both would be less expensive without pre-emptive isolation saving costs and unnecessary isolation of patients. When comparing the costs after this adjustment, culturing seems to be the preferred method since this was € 36,570 less expensive compared to PCR, even when in the culture method 66% more contacts (because test results were 2.3 days later known compared to PCR) were incorporated. An important drawback of abandoning pre-emptive isolation is the possible transmission of MRSA from non-isolated positive high risk inpatients towards other patients and/or HCWs. In our study this percentage was 26% and should not be ignored by policy and decision makers because outbreaks eventually entail high costs [10].

The present study also has several limitations. First, the SAB mortality rate was not adequately registered and as a consequence not available for analysis. Therefore, we used the SAB mortality rate of van Rijen et al. as proxy for our own SAB mortality rate [7]. Second, our benefits estimation could have been overestimated since ample evidence is available on the proportion of addition and replacement of current SAB rates with increasing MRSA prevalence rates, as well as on the Dutch hospital costs for a MRSA bacteraemia (in the present study we used the median hospital costs for patients with a MRSA bacteraemia of Cosgrove et al.) [16, 18]. In our study, we assumed (in line with van Rijen et al.) that in the worst-case scenario 100% of the MRSA cases were additional. However, a sensitivity analysis using the Kennemer Gasthuis data showed that in a 21% addition scenario (assuming a MRSA prevalence rate of 50%), the MRSA S&D policy in the Kennemer Gasthuis would already been cost effective. Probably, both addition and replacement coexist as was shown in the studies of Wyllie et al. and Wassenberg et al. [15, 18]. Third, in our benefits calculation we only included the costs and number of prevented MRSA bacteraemia patients, since this calculation can be performed accurately [7]. However, in practice, rising MRSA prevalence rates will also cause ‘less serious infections’ such as skin, soft tissue, post-surgical wound and catheter-related infections that also influence the benefits calculation (besides MRSA bacteraemia). When all these types of infections were taken into account, using the Kennemer Gasthuis data (including bacteraemia), 548 extra nosocomial MRSA infections were expected in the period between 2008–2013 (assuming 100% addition and 50% MRSA prevalence). Filice et al. calculated the median hospital costs for MRSA infected patients, independent of the site of infection at € 31,168 [19]. This would imply that the costs/savings in this situation would be € 17,080,064 (548 times € 31.168) which is on average € 2,846,677 euro per year. As a consequence the break-even point would be 1.7% (instead of 17.4%). However, these calculations are highly dependent of the used median hospitals costs for these infections and the assumed percentage of addition and replacement. These calculations must be interpreted with caution since not much evidence is available about the exact hospital costs and percentage of addition and replacement as mentioned earlier. Fourth, our study was performed in a single hospital in the Netherlands limiting the generalizability towards other countries that do not perform a comparable S&D policy or have higher prevalence rates. For example, the United Kingdom and United States of America differ largely from the Dutch S&D approach since MRSA guidelines do not include strict isolation measures and contact screening among unexpected MRSA positive patients [20, 21]. Furthermore, in some other European countries (Austria, Spain and Italy), no national MRSA control guidelines are available resulting in large differences in hospitals for controlling MRSA [22, 23]. However, Bootsma et al. suggested that a full or stepwise implementation of S&D in high prevalence countries may result in prevalence rates of <1% within 6–12 years which would make the policy highly cost effective [5].

One of the strengths of this study is the extensive costs description of the specific components for different patient groups (in- out- and dialysis patients), as well as the stratification of negative and positive test results that allows to study small changes for different patient groups (such as excluding pre-emptive isolation in the inpatient setting) and calculate costs. This enables a comparison of the MRSA S&D policy in the Kennemer Gasthuis with policies of other hospitals.

Our data showed that 72.1% of the MRSA positive patients were found unexpectedly and that only 3.3% of the high-risk patients carried MRSA. Consequently, 96.7% of the patients were unnecessarily isolated. We showed that excluding pre-emptive isolation is less expensive in spite of the assumed increase of the number of unexpected positive patients. For now, faster and cheaper diagnostics (for all MDROs) seem to have the greatest impact on costs and benefits of S&D policies. We advise other hospitals to record their infection control activities to study the MRSA costs and benefits as described in this study and expect that regional collaborations are increasingly important in infection control.

In conclusion, the MRSA S&D policy of the Kennemer Gasthuis is beneficial and should be continued to prevent the costs and deaths associated with increasing prevalence rates.
